# Nuclear transport of human cytomegalovirus tegument protein pp65 through nucleoplasmic reticulum

**DOI:** 10.1371/journal.ppat.1014224

**Published:** 2026-05-18

**Authors:** Maysa Azzeh, Mark F. Santos, Alexandra M. K. Yokomizo, Victoria Cha, Denis Corbeil, Aurelio Lorico

**Affiliations:** 1 Touro College of Osteopathic Medicine, New York, New York, United States of America; 2 College of Osteopathic Medicine, Touro University Nevada, Henderson, Nevada, United States of America; 3 Biotechnology Center (BIOTEC) and Center for Molecular and Cellular Bioengineering, Technische Universität Dresden, Dresden, Germany; 4 Tissue Engineering Laboratories, Medizinische Fakultät der Technischen Universität Dresden, Dresden, Germany; State University of New York Upstate Medical University, UNITED STATES OF AMERICA

## Abstract

Human cytomegalovirus (HCMV) is a widespread beta-herpesvirus that establishes lifelong infection and can cause severe disease in immunocompromised individuals as well as congenital abnormalities. While HCMV entry into fibroblasts is classically described as plasma membrane fusion, accumulating evidence indicates that a fraction of virions undergo endocytic uptake, notably via macropinocytosis, and traffic through the endosomal system. However, the mechanisms by which internalized viral components reach the nucleus are still being elucidated. Here, we investigated whether HCMV exploits type II nuclear envelope invaginations (NEIs), rare and discrete folds of the nuclear membrane that extend into the nucleoplasm, and the associated VAP-A–ORP3–Rab7 (VOR) complex to mediate nuclear delivery of viral components, a mechanism previously described for HIV-1. Using primary human foreskin fibroblasts (HFFs), we tracked the tegument protein pp65 and immediate-early proteins IE1/2 during early infection. We show that HCMV infection induces a rapid increase in NEI formation within the first hour of infection, accompanied by the accumulation of pp65 within Rab7 ⁺ endosomal structures that localize to NEIs. Pharmacological inhibition of the VOR complex with an ORP3-targeting drug significantly reduced NEI formation, decreased the association of pp65 with NEIs, and impaired its nuclear accumulation by approximately 2.5-fold. In contrast, inhibition of this pathway did not affect immediate-early gene expression at 24 hours post-infection. Functionally, disruption of the VOR complex resulted in a 3-fold reduction in viral replication, highlighting the contribution of this pathway to efficient infection. Together, these findings support a model in which HCMV tegument proteins, but not the viral genome, access the nucleus via a NEI/VOR-dependent trafficking route. This work identifies a previously unrecognized nuclear delivery pathway exploited by HCMV and suggests that targeting nuclear–endosomal communication may represent a novel antiviral strategy.

## Author summary

Human cytomegalovirus (HCMV) infects most people worldwide and can cause serious disease in newborns and individuals with weakened immune systems. To establish infection, viral components must reach the cell nucleus, but how this occurs after endocytic uptake remains unclear. In this study, we show that HCMV exploits specialized structures of the nuclear envelope, called type II nuclear envelope invaginations (NEIs), together with a host protein complex (VAP-A–ORP3–Rab7), to transport the viral tegument protein pp65 into the nucleus. Blocking this pathway reduces pp65 nuclear accumulation without affecting entry of the viral genome, indicating that different viral components use distinct routes to reach the nucleus. Importantly, disruption of this pathway also partly decreases viral replication, suggesting it contributes to efficient infection. These findings uncover a previously unrecognized intracellular trafficking route hijacked by HCMV and highlight nuclear-endosomal communication as a potential target for antiviral strategies.

## Introduction

Human cytomegalovirus (HCMV) is a widespread beta-herpesvirus that often remains asymptomatic in healthy populations, yet it triggers severe disease in immunocompromised individuals and represents the most common infectious source of congenital birth defects [1–5]. Understanding the complex interactions between HCMV and host cells is essential for developing effective vaccines and antiviral drugs.

The HCMV virions are large particles (150–300 nm in diameter) containing a linear, double stranded DNA genome within an icosahedral capsid, enveloped by a proteinaceous tegument and a lipid bilayer (reviewed in Ref. [6]). The infection process begins with a highly coordinated entry sequence, followed by the transport of viral components to the nucleus. The specific entry pathway is governed by the composition of envelope glycoproteins and their corresponding receptors on host cells [7–10]. Notably, the cell type in which the virus was propagated significantly influences its surface composition, thereby determining the subsequent entry mechanism [11]. While HCMV entry into epithelial and endothelial cells generally occurs via low-pH-dependent endocytosis [12], entry into fibroblasts typically involves direct fusion with the plasma membrane (reviewed in Ref. [7]).

However, research using the AD169 laboratory strain and clinical isolates has shown that viral particles can also enter fibroblasts via macropinocytosis, a non-selective, pH-independent bulk uptake process [13]. This uptake is dependent on various macropinocytosis-related factors, including dynamin (reviewed in Ref. [14]). During this process, virions colocalize with the fluid-phase marker and the small GTPase Rab5 in large vacuoles known as macropinosomes [13]. Within these vacuoles, the virions fuse with the endosomal membrane in a process that occurs independently of an acidic environment, releasing the viral capsid within the cytoplasm *en route* to the nuclear pore [13]. This process was monitored by following the tegument 150 kDa phosphoprotein (pp150), which remains tightly associated with the capsid [13].

In contrast, a transient co-localization of the pp65 and pp71 tegument proteins with Rab7 was observed [15]. This suggests that either a fraction of internalized virions bypasses early fusion to be trafficked into maturating macropinosomes and late endosomal compartments, or that specific tegument proteins remain associated with endosomal membranous structures following the fusion-mediated cytoplasmic release of the capsid [16]. Furthermore, these scenarios may represent a critical decision point: while these virions eventually escape these compartments to trigger lytic infection in fibroblasts, their continued trafficking through or entrapment within Rab7^+^ compartments in other cell types can facilitate the establishment of viral latency [15].

Although dispensable for viral replication [17], the major tegument pp65 protein rapidly accumulates in the nucleolus following internalization, a process mediated by its nuclear targeting signals [18]. As a multifunctional nuclear sub-compartment, the nucleolus provides a platform for pp65 to influence host cell processes, including modulation of nucleolar organization, cell cycle progression, and ribosomal RNA synthesis. Consistent with this, pp65 nucleolar localization has been linked to ongoing rRNA transcription, suggesting an indirect role in regulating nucleolar activity [19–21]. Beyond its structural role in viral architecture [17,22], pp65 has also been implicated in early immune evasion, notably by attenuating the host interferon response immediately after entry [23]. The nucleolar localization is significant due to its physical proximity to the nucleoplasmic reticulum [24]. Specifically, type II nuclear envelope invaginations (NEIs), i.e., deep inward foldings of both the inner and outer nuclear membranes, frequently terminate at or near the nucleolus [25].

We previously found that human immunodeficiency virus 1 (HIV-1) induces NEIs to facilitate nuclear translocation and subsequent productive infection [26]. This process involves late endosomes containing viral particles and a tripartite protein complex (referred as to VOR complex) composed of outer nuclear membrane protein VAP-A, the cytoplasmic oxysterol-binding protein ORP3, and late endosome-associated Rab7 [26,27]. Targeting the interaction of VAP-A–ORP3 with Rab7, for instance using small-molecule inhibitors directed at the ORP3 sterol-binding pocket, has been shown to block VOR complex assembly and impair both NEI formation and nuclear HIV-1 transfer [26,28].

Building on these findings, we investigated whether the tegument protein pp65 utilizes a similar pathway to bypass the crowded nucleoplasm and reach the sub-nuclear targets within nucleolus with high efficiency. We report that in infected fibroblasts, pp65 transits through NEIs on its way to the nuclear sub-compartment. Inhibition of the VOR complex significantly impaired both NEI formation and the nuclear translocation of pp65 without impacting the expression of immediate-early proteins IE1 and IE2. This suggests that HCMV utilizes distinct intracellular pathways to direct its genome and certain tegument components to their respective nuclear destinations.

## Results

### Viral tegument pp65 localizes to HCMV-induced NEIs

**Fig 1:**
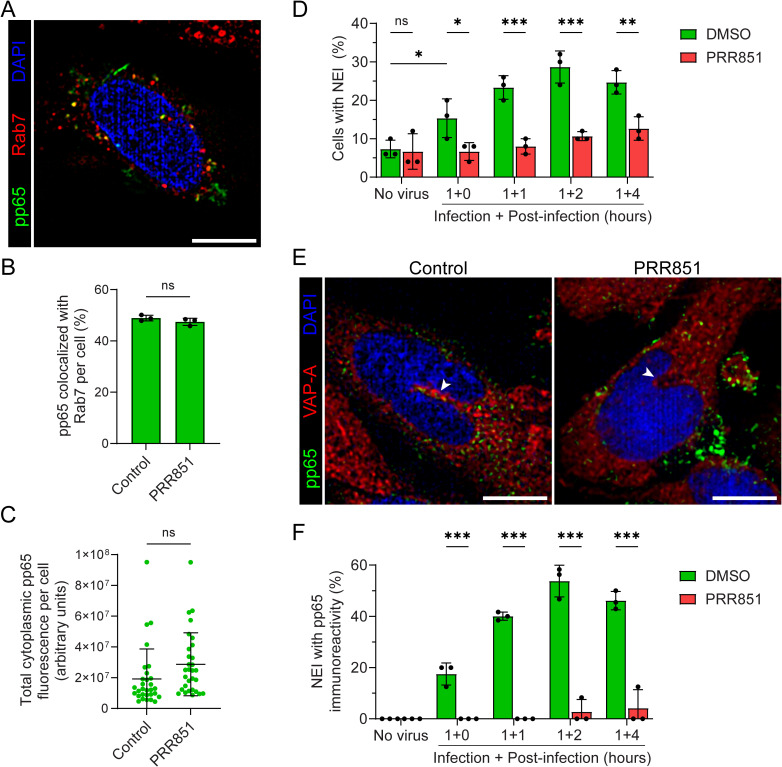
HCMV infection induces NEIs that recruit pp65 in a VOR-dependent manner. **(A-F)** HFFs were either directly infected with HCMV (MOI = 0.5) for 1 h (**A**) or pretreated with 10 µM PRR851 or DMSO (vehicle control) for 30 min prior to a 1 h infection **(B-F)**. Following infection, cells were washed and either processed immediately (**A-C**, **E**) or further incubated for 1, 2, or 4 hpi in the presence of PRR851 or DMSO as indicated **(D, F)**. MOCK-infected cells (no virus) served as negative controls **(D, F)**. Samples were processed for confocal microscopy after co-immunolabeling for the tegument protein pp65 (green) and the endosomal marker Rab7 (**A-C**, red) or VAP-A **(D-F)**. Cells were counterstained with 4′,6-diamidino-2-phenylindole (DAPI; blue). Several x-y optical sections were acquired throughout the cell volume to ensure comprehensive visualization (see S1 Fig). Single optical sections (0.25 μm) are displayed **(A, E)**. Following 1 h infection, partial co-localization of pp65 and Rab7 immunoreactivities was observed throughout the cytoplasmic compartment **(A)**, and quantified (**B**, mean ± **S.**D., 30 cells per independent experiment, n = 3). Pretreatment with PRR851 prior to infection impacted neither the co-localization (**B**) nor the cellular internalization of pp65 **(C)**. The number of HFFs harboring NEIs (visualized by VAP-A) significantly increased after 1 h of infection and remained through 4 hpi, a process effectively inhibited by PRR851 (**D**, mean ± **S.**D., 50 cells per independent experiment, n = 3). The presence of pp65 in VAP-A^+^ NEIs was blocked by PRR851 and quantified (**E**, **F**, mean ± **S.**D., 2-16 NEIs per independent experiment, n = 3). Arrowhead, NEI. Statistical significance: ns, not significant; *, *p* < 0.05; **, *p* < 0.01; ***, *p* < 0.001. Scale bar, 10 µm.

To characterize the intracellular trafficking of incoming HCMV particles, notably tegument pp65 proteins, we infected primary human foreskin fibroblasts (HFFs) with the AD169 strain (multiplicity of infection (MOI) = 0.5) for one hour. Afterward, we monitored the internalization of pp65 using indirect immunofluorescence and confocal microscopy. Based on earlier evidence that HCMV particles enter via macropinocytosis and are initially concentrated within large vacuoles positive for the small GTPase Rab5 [13], we co-immunolabeled our samples with small GTPase Rab7, a marker of late endosomes, to track this potential progression within the endosomal compartment. At early infection stages, pp65 immunoreactivity was observed as discrete cytoplasmic signals frequently overlapping with Rab7, suggesting its trafficking to maturing macropinosomes/late endosomes (Fig 1A). The quantitative analysis revealed a colocalization rate of 49% ± 1.1% (mean ± standard deviation (S.D.)) between pp65 and Rab7 (Fig 1B). These observations are consistent with the previously reported colocalization of HIV-1 integrase with Rab7 following HIV-1 infection [26]. This pp65–Rab7 colocalization, as well as the internalization of viral components, occurs independently of the VOR complex as no significant inhibitory effect was observed when cells were incubated with PRR851 drug, a small molecule directed against ORP3 that impedes VOR complex formation (Fig 1B and C) [26,29].

We next assessed whether early HCMV infection induces type II NEIs, as previously reported for HIV-1 [26,28]. These structures were morphologically defined as deep inward projections of the nuclear envelope into nucleoplasm [24]. These projections are positive for VAP-A, which localizes to both the endoplasmic reticulum and the outer nuclear membrane, and thus serves as a marker of these nuclear architectural features [30]. To accurately identify these discrete and relatively rare structures, given that most healthy cells exhibit none, while those with NEIs typically contain only one or two per nucleus [26,31], sequential x-y optical sections were acquired through the entire cell volume (see S1 Fig). In line with other healthy or uninfected cells, such as mesenchymal stromal cells and T cells, only 7.3 ± 2.3% of HFFs contained NEIs (Fig 1D). However, this number significantly increased to 15.3 ± 5.0% after 1 h HCMV infection and reached a peak of 28.7 ± 4.2% 2 h post-infection (hpi) (Fig 1D). Interestingly, the presence of PRR851 reverted the frequency of NEI-containing cells toward baseline levels during both initial 1 h infection period and the subsequent 1 and 2 hpi phases (Fig 1D). In agreement with the potential role of Rab7^+^ endosomal structures in this process, pp65 immunoreactivity was frequently observed adjacent to or within NEIs (Fig 1E). Quantification demonstrated a significant enrichment of pp65 signal in NEIs after 1 h after infection and over the the following 4 hpi, whereas PRR851 markedly reduced the proportion of pp65-containing NEIs (Fig 1F). Together, these findings suggest that early HCMV infection is associated with increased NEI formation and spatial enrichment of pp65 at these structures in a VOR complex-dependent manner.

### VOR complex inhibition blocks pp65 nuclear entry but not viral gene expression

**Fig 2:**
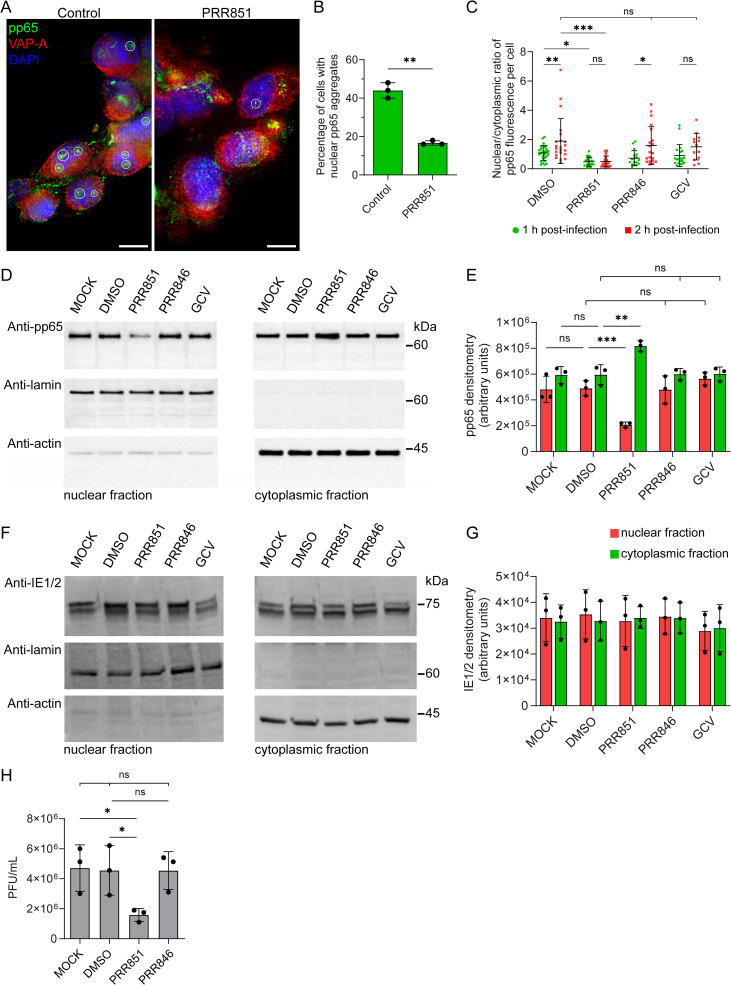
VOR complex inhibition prevents pp65 nuclear aggregation and reduces HCMV replication. **(A-C)** HFFs were pretreated with 10 µM PRR851 or DMSO (vehicle control) for 30 min prior to HCMV infection (MOI = 0.5) for 1 h**.** After inoculum removal, cells were incubated for an additional 1 (**C**) or 2 hpi (**A-C**) in the presence of treatments. Cells were then fixed, immunolabeled for pp65 (green) and VAP-A (red), and counterstained with DAPI (blue), and analyzed by confocal microscopy. Nuclear pp65^+^ aggregates (white circles) were detected in control cells but were significantly reduced in PRR851-treated cells; these were quantified (**A**, **B**, mean ± **S.**D., 50 cells per independent experiment, n = 3). **(C)** Cells were treated as in **(A)**, with the addition of an inactive PRR851 analogue (PRR846), or GCV. The nuclear-to-cytoplasmic pp65 ratio per cell was determined by quantifying pp65 immunoreactivities within the respective compartments (mean ± **S.**D., n = 13-28 cells). **(D-G)** Cells were either untreated (MOCK) or pretreated with PRR851, PRR846, GCV, or DMSO (vehicle control) as indicated. Following a 1 h infection and at 2 hpi (**D**, **E**) or 24 hpi **(F, G)**, nuclear and cytoplasmic fractions were prepared. Proteins in each fraction were analyzed by SDS-PAGE and immunoblotting for pp65 (**D**, **E**) and immediate-early proteins IE1/2 **(F, G)**. Lamin B1 and β-actin were used as nuclear and cytoplasmic markers, respectively. Molecular mass markers (kDa) are shown. The immunoreactivities of the proteins of interest in the nuclear fraction were quantified by densitometry (**E**, **G**; mean ± **S.**D., n = 3). **(H)** Viral titers in culture supernatants at 96 hpi in either MOCK-untreated cells or DMSO-, PRR851-, or PRR846-treated cells were determined by standard plaque assay. Plaques were quantified 10–14 days post-infection via inverted microscopy to calculate plaque-forming units per milliliter (PFU/mL). Images represented single optical sections (0.25 μm). Scale bars, 10 µm. Statistical significance: ns, not significant; *, *p* < 0.05; **, *p* < 0.01; ***, *p* < 0.001.

To determine whether the nuclear entry of HCMV components depends on the VOR complex, we infected HFFs for 1 h and, at 2 hpi, examined the intracellular distribution of pp65 using confocal microscopy, both in the presence or absence of PRR851. In the control, pp65 immunoreactivity accumulated within the nuclear compartment, forming few nuclear aggregates per cell (Fig 2A). These pp65^+^ aggregates mirror previous findings showing the early pp65 accumulation in the nucleolus, where it may interact with nucleolin [20,21]. Multifunctional protein nucleolin plays critical roles in various cellular pathways far beyond its traditional functions in ribosomal assembly and maturation (reviewed in Ref. [32]). In sharp contrast, these pp65^+^ aggregates were significantly reduced in the presence of PRR851 (Fig 2A and 2B). These observations were consistent with an overall reduction of nuclear pp65 immunoreactivity as detected by the nuclear-to-cytoplasmic ratio of pp65 immunoreactivity at 1 or 2 hpi (Fig 2C). Conversely, treatment with an inactive PRR851 analogue (PRR846) [28,29] or the antiviral drug ganciclovir (GCV) did not produce this effect. Under these conditions, we did not observed a reduction of the total pp65 (nuclear + cytoplasm) compared to the control samples (DMSO) (see S1 Data). The lack of action of GCV could be explained by its mechanism, as it inhibits viral DNA replication at later stages of infection [33]. The PRR851-dependent inhibition of the nuclear transfer of pp65 was observable until 48 hpi, but declined gradually afterward (S2 Fig).

These data were further validated biochemically by fractionating the nuclear and cytoplasmic compartments. The distribution of pp65 levels was analyzed by immunoblotting, using Lamin B1 and actin as nuclear and cytoplasmic loading control markers, respectively. Consistent with our microscopy findings, we observed a significant shift in the distribution of pp65 between the two fractions in the presence of PRR851, but not with the two other compounds (Fig 2D and 2E).

Interestingly, this VOR-dependent inhibition was specific to pp65 and did not extend to the nuclear entry of the HCMV genome. Accordingly, the expression levels of IE1/2 proteins at 24 hpi, a time point sufficient for viral genome entry, transcription, translation, and the subsequent nuclear localization of these proteins [34–36] were not impacted by PRR851 treatment (Fig 2F and 2G). These results indicate that the inhibition of NEI formation and the VOR complex does not prevent immediate-early protein expression. Altogether, these findings suggest that the nuclear entry of the HCMV genome occurs through a pathway distinct from the NEI/VOR-dependent transport used by pp65.

### VOR complex inhibition reduces viral replication

Finally, we assessed whether inhibition of pp65 nuclear entry affects viral replication using a plaque assay. To this end, HFFs were pretreated for 30 min with PRR851 or control compounds prior to infection with HCMV (MOI = 0.5) for 1 h. Viral supernatants were collected at 96 hpi and analyzed. HCMV replication in PRR846- and DMSO-treated cells was comparable to that in untreated controls, whereas PRR851 treatment resulted in an approximately three-fold reduction in viral plaque formation (Fig 2H).

Altogether, these findings indicate that HCMV relies, at least in part, on VOR-dependent NEI trafficking for efficient delivery of the pp65 tegument protein to the nucleus. Given that pp65 is dispensable for viral replication [17], the inhibitory effect of PRR851 likely reflects impaired nuclear delivery of additional viral components and/or disruption of downstream processes required for optimal viral propagation.

## Discussion

Here, we report a novel intracellular route utilized by the HCMV tegument protein pp65 to reach HFF nuclei shortly after infection. This pathway involves the formation of type II NEIs and the incorporation of late endosomal structures therein, a process mediated by the molecular complex named VOR [27,30]. We have previously termed these dual organelles “spathasomes” [31]. These observations provide a potential explanation for the previously reported differential subnuclear localization of pp65 [19] and its rapid accumulation in the nucleolus [20,21]. Notably, a similar pathway has been shown to be critical for productive HIV-1 infection in T-lymphocytes [26]. However, pharmacological inhibition of the VOR complex using the small-molecule inhibitor PRR851, developed in our laboratory [26,29], did not impair IE1/2 protein expression or overall HCMV replication. These findings support the existence of divergent intracellular routes utilized by pp65 and the HCMV genome to access the nuclear compartment.

Given that the VOR complex involves the small GTPase Rab7, our data suggest that a fraction of internalized pp65 traffics through late endosomal compartments, which is consistent with previous observations [16]. In contrast, capsids and the capsid-associated tegument proteins, such as pp150, are thought to escape from maturing endosomes prior to docking at nuclear pores [13]. We therefore propose that pp65 (or potentially pp71) remains transiently associated with the cytoplasmic side of the endosomal membrane before accessing NEIs. Within these dual structures, the release of pp65 into confined cytoplasmic channels may facilitate its rapid transfer to the nucleus and subsequent accumulation (or condensation) in nucleolar-associated domains via the nuclear import mechanism. This model is consistent with HCMV entry into fibroblasts via a pH-independent mechanism, despite the involvement of endocytic uptake pathways [7,13], supporting the notion that membrane fusion and downstream intracellular trafficking can be mechanistically uncoupled. A key viral advantage of the spathasome nuclear pathway is that late endosomal compartments may protect certain viral components, such as tegument proteins, from cytoplasmic degradation, while simultaneous avoiding lysosomal maturation. Consistent with this, we previously observed the exclusion of the lysosomal marker lysosome-associated membrane protein 1 from type II NEIs [26].

Our data do not exclude the possibility that a fraction of HCMV enters cells via direct fusion at the plasma membrane [37] or evades fusion within the early endosomal compartment, thereby continuing its trafficking into late endosomal structures. Additional factors, such as extracellular vesicles (EVs), may also contribute to infection. Indeed, while pp65 is a well-established component of infectious virions, it has also been detected in non-infectious particles, including large, tegument-rich structures known as dense bodies, particularly following viral propagation in fibroblasts [38–41]. Upon uptake, these large subviral EVs may alter the composition of host cells [42] or enhance interferon-beta responses [43]. Interestingly, we previously observed that EV cargoes can access the nucleus of recipients cells via a mechanism sensitive to VOR complex inhibitors [29]. These findings highlight a similarity in the cellular uptake and nuclear targeting of cargoes from extracellular particles, whether viral or EV-derived [27]. These various scenarios, which are not mutually exclusive, warrant further examination. The same applies to the infection of epithelial and endothelial cells, which is known to occur via endocytosis in an acidic environment, specifically in late endosomes [12]. Although the inhibition of the VOR complex in HFFs led to only a 3-fold reduction in viral replication as evaluated by a standard plaque assay, the impact might be more significant in other cell types. Moreover, the incorporation of late endosomes into NEIs may also be relevant to other viruses such as the oncogenic Epstein-Barr and Kaposi’s sarcoma virus.

Finally, it is important to note that, to our knowledge, the formation of type II NEIs during the early stages of HCMV infection has not been previously reported in fibroblasts [44]. The lack of prior documentation may be explained by the fact that these nuclear structures are discrete and generally rare in healthy cells, typically occurring only once to thrice per cell [24,31], unlike in cancer cells where they are more prevalent [45]. Consequently, identifying them requires a serial series of confocal x-y optical sections, as demonstrated here using VAP-A immunostaining. In contrast, type I NEIs, i.e., invaginations involving solely the inner nuclear membrane, are typically observed during the later stages of HCMV infection [44]. Of note, a structure similar to type II NEIs, termed the “Tegusome” has been described in thymocytes infected with human herpesvirus 6 [46]. Whether tegusomes are functionally identical to the type II NEIs described here warrants further investigation.

In conclusion, this study identified a novel, VOR-mediated, type II NEI-dependent pathway for rapid HCMV tegument protein pp65 nuclear localization, distinct from the pathway used by the viral genome. By utilizing late endosomal trafficking to access the nucleus, the virus forms ‘Spathasome/Tegusome-like” structures that protect cargo from degradation, representing a potential new paradigm in beta-herpesvirus infection.

## Materials and methods

### Cell culture

Primary human foreskin fibroblasts (HFFs; ATCC-SCR-1041) were maintained in Dulbecco’s Modified Eagle Medium (DMEM; ATCC 30–2002) supplemented with 10% fetal bovine serum and 100IU/ml of Penicillin and Streptomycin. Cultures were incubated at 37°C in a humidified atmosphere containing 5% CO_2_. Cells used for all experiments were between passages 16–18 to ensure consistency.

### HCMV strain and production

The HCMV laboratory strain AD169 (ATCC-VR-538) was propagated in HFFs. Viral stocks were generated by infecting confluent monolayers at a low multiplicity of infection (MOI) until full cytopathic effect was observed. Supernatants were clarified, aliquoted, and stored at −80°C. Viral titers were determined by plaque assay.

### Plaque assay

To quantify infectious virus, HFFs were seeded in 24-well plates and infected with serial 10-fold dilutions (10^−3^ to 10^−7^) of viral supernatant collected from HCMV-infected cells. Following a 2 h of adsorption period, the inoculum were removed and replaced with complete DMEM containing 0.25% agarose to restrict viral spread. Plates were incubated at 37°C until plaques were visible (10–14 days). Plaques were visualized using an inverted microscope and counted. Titers were calculated as plaque-forming units per milliliter (PFU/mL), accounting for the dilution factor and volume of inoculum. Formula is below:


$$\begin{array}{c} plaque-forming\;units\;\left(\frac{PFU}{mL}\right)=\frac{number\;of\;plaques}{dilution\;factor\times volume\;of\;inoculum\;(mL)} \end{array}$$

### Infection and drug treatment

HFFs were seeded onto 8-well chamber slides (80826, iBidi). Cells were either MOCK-treated or pretreated for 30 min with 10 µM of drugs (PRR851, PRR846, or ganciclovir (GCV)), or vehicle (DMSO). Subsequently, cells were infected with HCMV at an MOI of 0.5 for 1 h in the continuous presence or absence of drugs. Following infection, cells were washed three times with PBS and cultured in fresh medium with or without drugs for 1, 2, 4, 24, and 72 hpi. Samples were analyzed either directly at the end of the 1 h infection, or after post-infection.

### Immunocytochemistry and confocal microscopy

Infected cells were washed, fixed in 4% paraformaldehyde for 15 min, and permeabilized with 0.2% Tween-20 for 15 min. Immunolabeling was performed using primary antibodies (Abs) against HCMV pp65 (HCMV UL83-CH12, VS-P1205, SeraCare), VAP-A (A304-366A, Fortis Life Sciences), and Rab7 (ab137029, Abcam). Fluorophore-conjugated secondary Abs were applied for 30 min: anti-mouse Alexa Fluor488 (A-11017, Thermo Fisher Scientific) and/or anti-rabbit Alexa Fluor647 (A-21246, Thermo Fisher Scientific). Nuclei were counterstained with DAPI. Samples were imaged in PBS by structured illumination microscopy using a Nanoimager confocal fluorescence microscope (ONI, Oxford, UK) equipped with a 100x oil-immersion objective. Identical acquisition settings were maintained across all experimental conditions. Quantitative image analysis was performed using Fiji software [47]. Colocalization was quantified by calculating the percentage of pp65 signal overlapping with the Rab7 signal per cell. NEIs were defined as VAP-A-positive inward projections continuous with the nuclear rim; cells were scored as NEI-positive if at least one such structure was identified. Nuclear-to-cytoplasmic pp65 fluorescence ratios were calculated by measuring the mean fluorescence intensity (MFI) within manually DAPI-defined nuclear and cytoplasmic regions of interest (ROIs). Note that all individual optical sections (0.25 μm) were analyzed.

### Nuclear and cytoplasmic protein extraction and immunoblotting

Nuclear and cytoplasmic fractions of infected cells were prepared using NE-PER™ Nuclear and Cytoplasmic Extraction Reagents (PI78833, Thermo Fisher Scientific) according to the manufacturer’s protocol [26]. Proteins were transferred to nitrocellulose membranes (88018, Thermo Fisher Scientific) and blocked in 1% bovine serum albumin in PBS for 1 h at room temperature (RT). Membranes were incubated overnight at 4°C with primary Abs against HCMV pp65 (see above), HCMV IE1/2 (VS-P1215, SeraCare), Lamin B1 (sc-374015, Santa Cruz Biotechnology), or β-actin (A2228, Sigma-Aldrich). Lamin B1and β-actin were used as a nuclear marker and as a cytoplasmic marker, respectively, to monitor the fraction purity. After washing, membranes were incubated with Alexa Fluor488-conjugated secondary Abs for 30 min at RT. Fluorescent signals were visualized and quantified using the iBright FL1000 imaging system (Thermo Fisher Scientific). Densitometric analysis was performed using iBright analysis software.

### Statistical analysis

All experiments were carried out at least in triplicate. Data are presented as mean ± standard deviation (S.D.). Statistical significance was determined using a two-tailed Student’s t-test or two-way ANOVA with Bonferroni multiple comparisons test. Comparisons with *p* values < 0.05 were considered significant. All graphs were created using GraphPad Prism 9.

## Supporting information

S1 Data All individual values shown are included in the Data Source. (XLSX)

S1 Fig Serial optical sections showing NEI morphology and pp65 localization. HFFs were infected with HCMV (MOI = 0.5) for 1 h and processed for confocal microscopy following co-immunolabeling for VAP-A (red) and the tegument protein pp65 (green). To ensure comprehensive visualization of the nuclear architecture, 36 x-y optical sections (0.25 μm) were acquired from the bottom to the top of the cell. Discrete VAP-A-positive NEIs appear as inward projections continuous with the nuclear rim and can be traced across multiple optical planes (white arrows). The pp65 immunoreactivity is observed adjacent to or sequestered within these structures in specific optical sections. Nu, nucleus; Cy, cytoplasm. Scale bar, 10 µm.
(PDF)

S2 Fig Time-course analysis of pp65 nuclear localization inhibition by PRR851 throughout HCMV infection. HFFs were pretreated for 30 min with 10 µM of the indicated compounds (PRR851, PRR846, or GCV), or DMSO vehicle, followed by HCMV infection (MOI = 0.5) for 1 h. After inoculum removal, cells were incubated in the presence of treatments for an additional 1, 2, 24, 48, or 72 hpi (see Materials and Methods). Cells were fixed and co-immunolabeled for the tegument protein pp65 and VAP-A (to delineate the nuclear membrane). The nuclear-to-cytoplasmic pp65 ratio per cell was determined by quantifying pp65 immunoreactivities within the respective compartments using confocal microscopy. Data represented mean ± S.D. (n = 30–55 cells per condition). Statistical significance: ns, not significant; *, *p* < 0.05; **, *p* < 0.01; ***, *p* < 0.001.
(PDF)
