# Supplementary figures and images for: Nuclear transport of human cytomegalovirus tegument protein pp65 through nucleoplasmic reticulum
Source: PLoS Pathog. 2026 May 18;22(5):e1014224. doi: 10.1371/journal.ppat.1014224 (PMC13215604; doi:10.1371/journal.ppat.1014224)

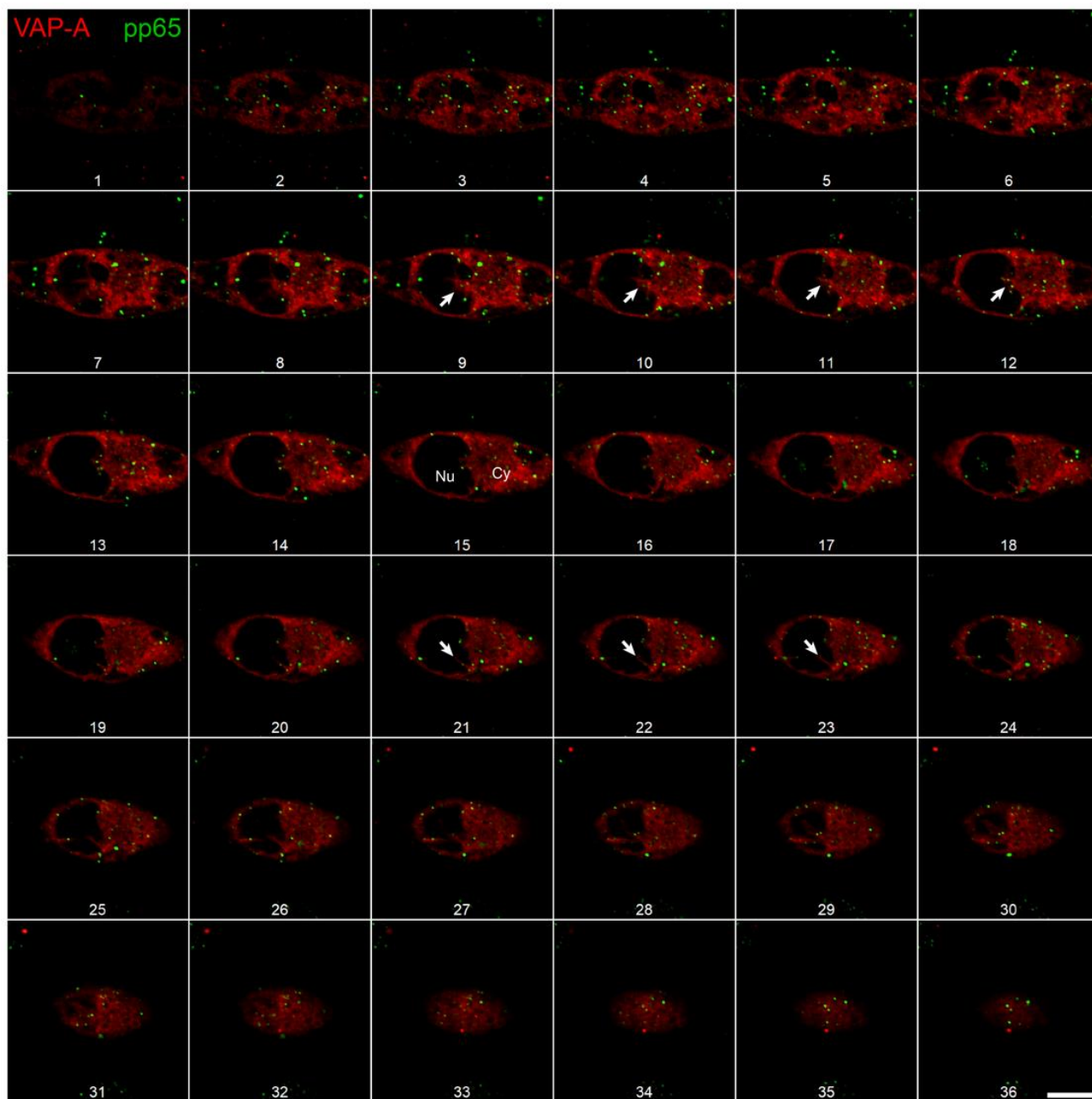

Supplement: S1 Fig — HFFs were infected with HCMV (MOI = 0.5) for 1 h and processed for confocal microscopy following co-immunolabeling for VAP-A (red) and the tegument protein pp65 (green). To ensure comprehensive visualization of the nuclear architecture, 36 x-y optical sections (0.25 μm) were acquired from the bottom to the top of the cell. Discrete VAP-A-positive NEIs appear as inward projections continuous with the nuclear rim and can be traced across multiple optical planes (white arrows). The pp65 immunoreactivity is observed adjacent to or sequestered within these structures in specific optical sections. Nu, nucleus; Cy, cytoplasm. Scale bar, 10 µm. (PDF) [file ppat.1014224.s002.pdf]

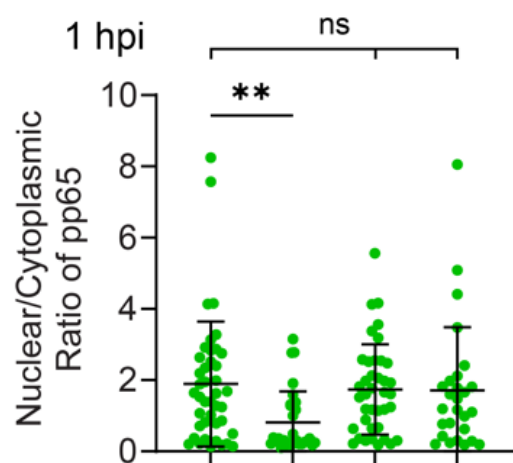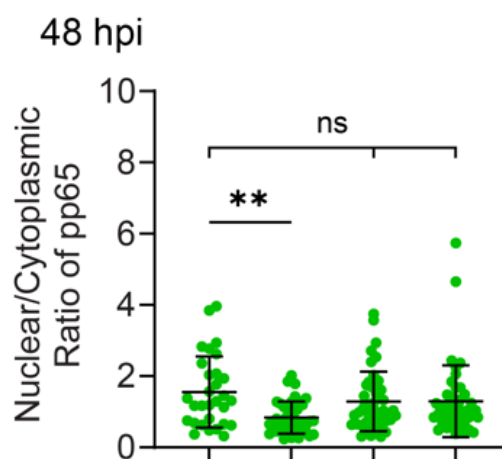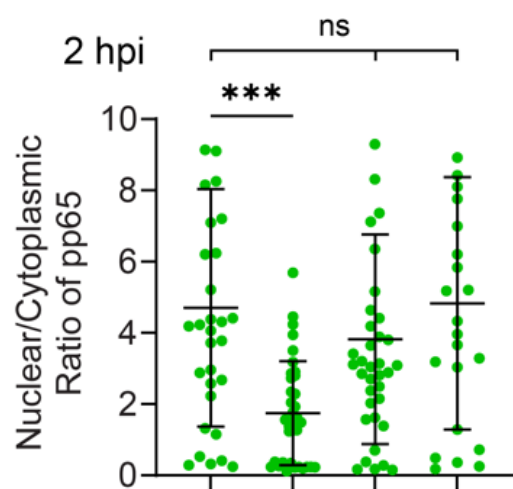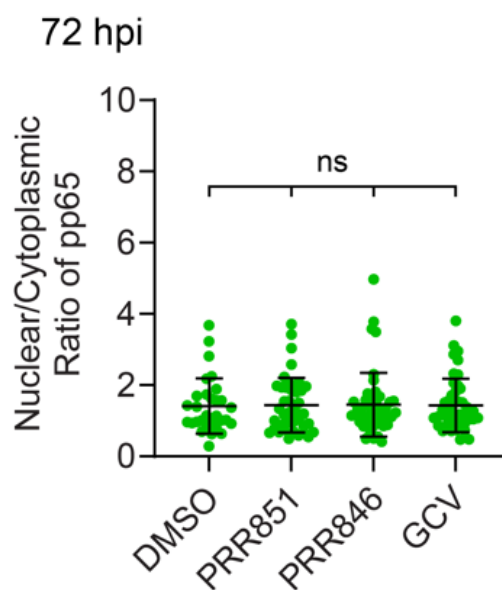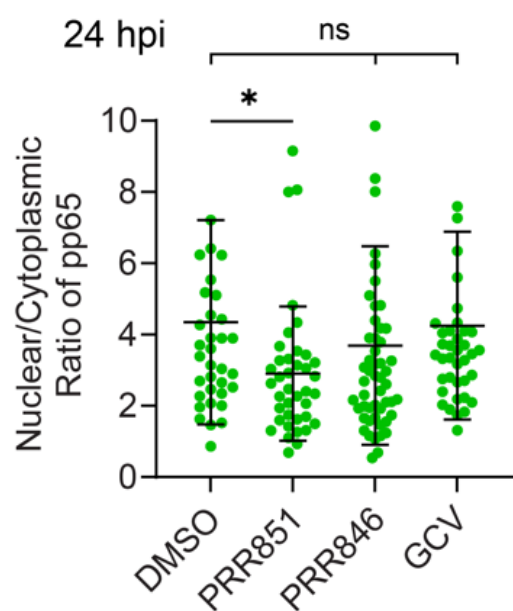

Supplement: S2 Fig — HFFs were pretreated for 30 min with 10 µM of the indicated compounds (PRR851, PRR846, or GCV), or DMSO vehicle, followed by HCMV infection (MOI = 0.5) for 1 h. After inoculum removal, cells were incubated in the presence of treatments for an additional 1, 2, 24, 48, or 72 hpi (see Materials and Methods). Cells were fixed and co-immunolabeled for the tegument protein pp65 and VAP-A (to delineate the nuclear membrane). The nuclear-to-cytoplasmic pp65 ratio per cell was determined by quantifying pp65 immunoreactivities within the respective compartments using confocal microscopy. Data represented mean ± S.D. (n = 30–55 cells per condition). Statistical significance: ns, not significant; *, p < 0.05; **, p < 0.01; ***, p < 0.001. (PDF) [file ppat.1014224.s003.pdf]
